# Supplementary figures and images for: Three-Dimensional Modelling of Indexed Papillary Muscle Displacement in Patients Requiring Mitral Valve Surgery Using Four-Dimensional Echocardiography Variables
Source: J Clin Med. 2024 Dec 10;13(24):7503. doi: 10.3390/jcm13247503 (PMC11677786; doi:10.3390/jcm13247503)

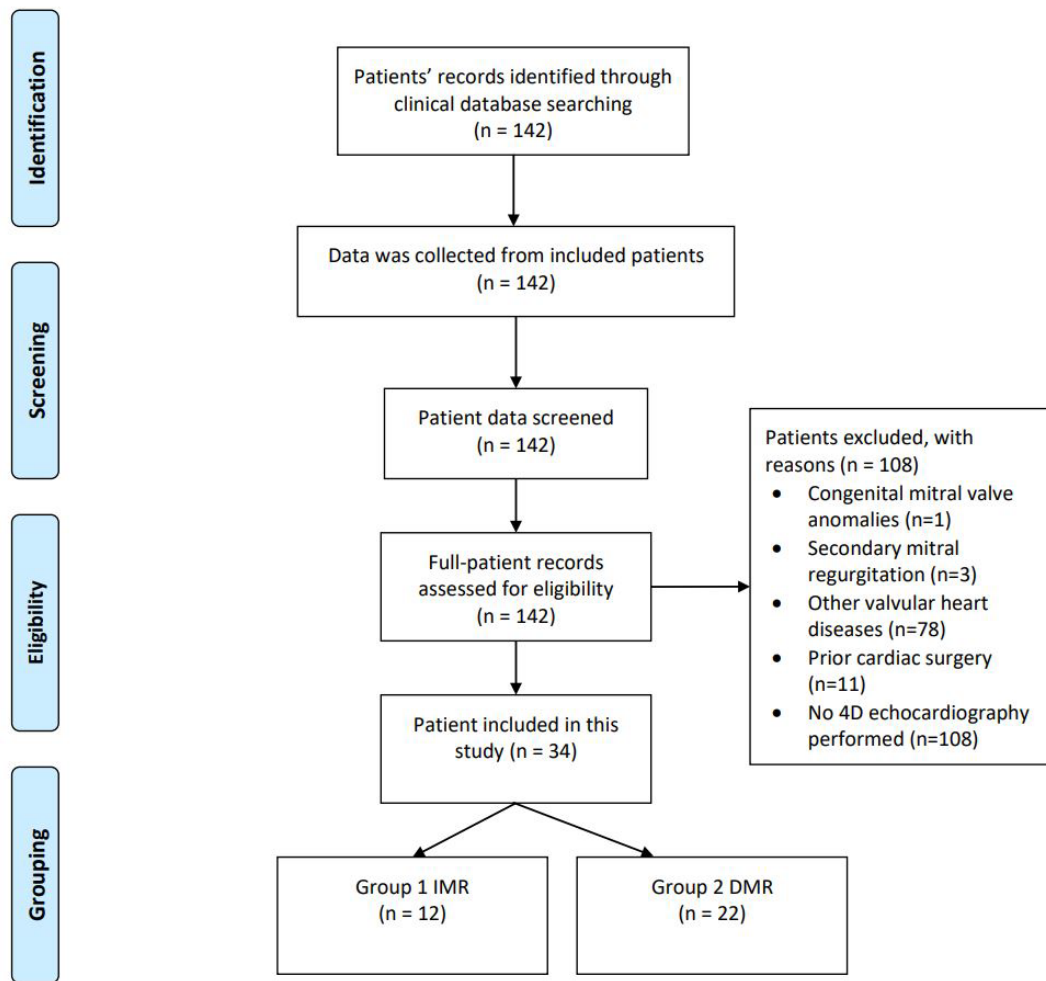

**Figure S1:** Study flowof diagram.

Supplement: Supplementary file 1 [file jcm-13-07503-s001.zip › jcm-3293487-supplementary.pdf]
